# The interaction between Mediterranean diet and intestinal microbiome: relevance for preventive strategies against frailty in older individuals

**DOI:** 10.1007/s40520-024-02707-9

**Published:** 2024-03-06

**Authors:** Andrea Ticinesi, Antonio Nouvenne, Nicoletta Cerundolo, Alberto Parise, Pedro Mena, Tiziana Meschi

**Affiliations:** 1 Department of Medicine and Surgery, University of Parma, Via Antonio Gramsci 14, 43126 Parma, Italy; 2 Microbiome Research Hub, University of Parma, Parma, Italy; 3 Geriatric-Rehabilitation Department, Azienda Ospedaliero-Universitaria Di Parma, Parma, Italy; 4 Human Nutrition Unit, Department of Food and Drugs, University of Parma, Parma, Italy

**Keywords:** Frailty, Dementia, Sarcopenia, Malnutrition, Healthy diet

## Abstract

Age-related changes in intestinal microbiome composition and function are increasingly recognized as pivotal in the pathophysiology of aging and are associated with the aging phenotype. Diet is a major determinant of gut-microbiota composition throughout the entire lifespan, and several of the benefits of a healthy diet in aging could be mediated by the microbiome. Mediterranean diet (MD) is a traditional dietary pattern regarded as the healthy diet paradigm, and a large number of studies have demonstrated its benefits in promoting healthy aging. MD has also a positive modulatory effect on intestinal microbiome, favoring bacterial taxa involved in the synthesis of several bioactive compounds, such as short-chain fatty acids (SCFAs), that counteract inflammation, anabolic resistance, and tissue degeneration. Intervention studies conducted in older populations have suggested that the individual response of older subjects to MD, in terms of reduction of frailty scores and amelioration of cognitive function, is significantly mediated by the gut-microbiota composition and functionality. In this context, the pathophysiology of intestinal microbiome in aging should be considered when designing MD-based interventions tailored to the needs of geriatric patients.

## Introduction

The intestinal microbiome is the ensemble of microorganisms, predominantly bacteria, living in the gastrointestinal lumen and establishing physiologic interactions with the human body, as well as their theater of activity including a whole set of molecules related to gut and host physiology [1]. In healthy adult subjects, an equilibrium between bacterial species with purported health-promoting properties and opportunistic pathogens is generally present, while significant differences in gut microbial communities can be detected among different subjects [1, 2]. This inter-individual variability depends on a large number of factors, including host genetics, environmental exposures, lifestyle and physiologic status [3, 4]. Diet is perhaps the most influential of these factors, as suggested by metagenome-wide association studies [4, 5].

The process of aging implies a certain degree of disruption of the equilibrium between beneficial, neutral and potentially pathogenic bacteria [6], especially after 70 years old, partly as a consequence of aging of the gastrointestinal and immune system [7], and partly as the result of disease, exposure to drugs, and change of diet and mobility [8]. This imbalance of intestinal microbial communities can negatively influence several aspects of the host physiology and is defined as dysbiosis. Although dysbiosis can occur also in earlier stages of the human life, it is consistently involved in the pathogenesis of several age-related diseases and syndromes and thus increasingly regarded as one of the fundamental pathogenetic drivers of aging [9].

Dysbiosis does not only imply a change in the composition of the microbiome, with increased representation of opportunistic pathogens at the expense of taxa with purported health-promoting activity, but it is also associated with a different microbiome functionality, causing changes in the release of physiologically active compounds [10]. In the last decade, several experimental and clinical studies have suggested that age-related gut-microbiota alterations can negatively influence the pathogenesis of many diseases and conditions with high prevalence in geriatric patients [11], including dementia [12], sarcopenia [13], type 2 diabetes [14], hypertension, and other cardiovascular diseases [15].

In older subjects, inter-individual differences in intestinal microbiota composition are also emphasized in comparison with subjects under 70 years of age [16]. As such, fit individuals who reach extreme ages of life, such as centenarians and supercentenarians, may maintain an intestinal microbiome structure more similar to the one of adult subjects, with good representation of bacterial taxa with beneficial modulatory properties for the body functioning, such as anti-inflammatory and pro-anabolic action [17]. Conversely, the deepest levels of dysbiosis are generally observed in frail multimorbid subjects [18, 19].

These circumstances suggest that maintenance of a good equilibrium in intestinal microbiome should be a goal for promoting successful aging [20]. The administration of live bacteria (i.e., probiotics) or functional components (i.e., prebiotics) and foods has shown limited effectiveness on microbiome structure and clinical endpoints in older individuals, although few studies are available to date [21–23]. Dietary interventions, instead, have been emphasized as promising strategies to counteract dysbiosis by inducing generalized and durable rearrangements on microbiome composition and function [24]. Mediterranean diet (MD), in particular, has emerged as the healthy diet paradigm and has been associated with a wide range of beneficial effects on primary and secondary prevention of several non-communicable diseases [25, 26].

In this review, we summarize the current evidence on the interactions between MD and gut microbiome, and their importance for mitigating the pathophysiological processes associated with aging and some of the most relevant age-related conditions. First, we consider the effect of MD on the microbiome of adult subjects; then, we explore the impact of MD on older adults; and last, we comment on the main underlying mechanisms behind the anti-aging effects of MD on the gut microbiome.

## Association between Mediterranean diet and microbiome in adult subjects

### Observational studies

MD, acknowledged by UNESCO as Intangible Cultural Heritage of Humanity in 2010, is a traditional dietary pattern implying daily consumption of plant foods, including cereals, fruit, vegetables and legumes with olive oil as the main source of added fat, and moderate consumption of fish, seafood, eggs, poultry and milk or dairy products. Read meat and unprocessed sugars, which represent important components of modern Western diets, are generally present in limited amounts in MD [27], as well as parsimonious consumption of wine during mealtimes. Apart from its mere composition, MD also implies socialization and conviviality during meals, seasonality and biodiversity in dietary choices, frequent consumption of traditional and local food products and, overall, a healthy lifestyle with a good balance between physical activity and rest [27].

Adherence to MD pattern can be measured in each individual through specific scores validated by the scientific literature. These scores are generally based on the analysis of semi-quantitative food-frequency questionnaires and on the identification of the frequency of consumption of key foods for MD [28]. Despite the limitations of this approach, including the possibility of recall bias, population-based studies conducted in different countries have shown a correlation between MD adherence scores and fecal microbiome composition [29–35]. The results of these studies are summarized in Table 1.

**Table 1:** Overview of the main findings of the observational studies investigating the relationship between Mediterranean diet and gut microbiome in adult subjects

Main author, year, reference	Participants	Main microbial biomarkers of MD	Main microbial biomarkers of Western diet	Other key findings
Gutiérrez-Díaz 2016 [29]	Thirty-one adults not reporting chronic diseases	*Bacteroidetes* *Prevotellaceae* *Prevotella*	*Firmicutes* *Lachnospiraceae*	MD adherence was associated with increased fecal propionate and butyrate
De Filippis 2016 [30]	One hundred and fifty-three adults habitually following omnivore, vegetarian or vegan diets	*Lachnospira* *Prevotella*	*Ruminococcus* *Streptococcus*	MD adherence was associated with representation of fiber-degrading bacteria and increased fecal SCFA levels Omnivore diet implied higher levels of TMAO
Mitsou 2017 [31]	One hundred and sixteen adult volunteers in good health	*Bifidobacteria* *Candida albicans*	*Bacteroides* *Escherichia coli*	MD adherence was associated with increased production of SCFAs and with lower burden of gastrointestinal symptoms
Gallé 2020 [32]	One hundred and forty university students	*Lachnospira* *Lactobacillus* *Lactococcus*	*Ruminococcus* *Oscillospira* *Paraprevotella*	Microbiota composition was more influenced by habitual physical activity and BMI than MD adherence scores
Wang 2021 [33]	Three hundred and seven male health professionals aged 45 or older	*Faecalibacterium prausnitzii* *Eubacterium rectale* *Bacteroides cellulosilyticus*	*Ruminococcus torques* *Clostridium leptum* *Collinsella*	MD adherence was associated with increased plant polysaccharide degradation potential and SCFA synthesis The association between MD adherence and blood biomarkers of cardiometabolic risk was mediated by *Prevotella copri* abundance
Turpin 2022 [34]	Two thousand two hundred and eighty nine healthy relatives of subjects with Chron’s disease	*Lachnospira* *Faecalibacterium* *Clostridium* *Veillonella*	*Ruminococcus* *Dorea* *Actinomyces*	*Clostridium, Parvimonas* and *Dialister* were the main taxa mediating the anti-inflammatory effect of diet on the intestinal mucosa
Rosés 2021 [35]	Three hundred and sixty adults with different weight (normal, overweight or obese)	*Bifidobacterium animalis* *Roseburia faecis* *Flavonifractor plautii* *Ruminococcus bromii*	*Eubacterium saphenum* *Succinivibrio dextrinosolvens* *Gordonibacter pamelaeae*	MD adherence was significantly associated with the abundance of several bacterial taxa able to synthetize SCFAs, particularly butyrate

*MD* Mediterranean Diet; *SCFA* Short-Chain Fatty Acids; *TMAO* Trimethylamine-*N*-oxide; *BMI* Body Mass Index

Interestingly, in most studies, MD adherence was significantly associated neither with biodiversity, that is, species richness, nor with the overall composition of the intestinal microbial community [29, 33, 34]. Instead, MD seemed more associated with increased representation of bacterial taxa with purported health-promoting activities and metabolic function of bacteria, like *Lachnospira, Prevotella,* bifidobacteria, *Faecalibacterium prausnitzii, Eubacterium rectale* [29–35]. Conversely, *Ruminococcus, Oscillospira, Escherichia coli* and other members of *Enterobacteriaceae* were the main microbial taxa associated with Western-style diets [32, 34].

At functional level, MD adherence was associated with different metabolic signatures in blood, urine and fecal samples [36]. In particular, it was associated with increased microbial synthesis of short-chain fatty acids (SCFAs), including acetate, propionate and butyrate, which exhibit an overall anti-inflammatory and pro-anabolic function favoring insulin sensitivity [29–31, 33]. Bacterial taxa with known capacity of synthetizing SCFAs, such as *F. prausnitzii*, *Butyrivibrio,* and *Roseburia*, seem to play an important role as mediators of the beneficial metabolic effects of MD [37]. High fiber intake, which is a key component of MD, is in fact able to stimulate the representation of these taxa in intestinal microbial communities, while fibers themselves represent the biochemical substrates for SCFA synthesis [38].

MD adherence was also associated with reduced synthesis of trimethylamine-*N*-oxide (TMAO) [30, 32], an emerging marker of cardiovascular risk synthetized with the contribution of the gut microbiota [39]. According to a study conducted on 307 male adults, *Prevotella copri* also resulted as a key mediator of the beneficial effects of MD adherence on biomarkers of cardiometabolic risk, including serum levels of C-reactive protein (CRP), total cholesterol, triglycerides, and glycated hemoglobin [33]. Microbiome mediation in the relationship between MD adherence and reduction of inflammation was also confirmed in groups of patients with chronic inflammatory conditions, such as human immunodeficiency virus (HIV) infection [40], or at risk of inflammatory bowel disease (IBD) [34].

### Intervention studies

MD intervention is generally assumed to modify gut-microbiome composition and function not only in subjects with chronic diseases (Table 2), but also in healthy subjects [41–43]. The magnitude and the characteristics of these changes, however, are inconsistent across studies.

**Table 2:** Summary of the main findings of randomized controlled trials testing the effects of Mediterranean diet on gut microbiome in subjects with chronic illness

Reference	Participants	Study arm	Comparison arm	Duration of intervention	Effects on gut microbiome	Effects on other physiologic parameters	Other key findings
Haro 2016 [46]	Twenty obese subjects	MD	LFHCC diet	1 year	↑ *Roseburia, Oscillospira, Parabacteroides distasonis* ↓ *Prevotella, Faecalibacterium prausnitzii*	↑ Insulin sensitivity index Different urinary metabolomic profile	LFHCC diet also induced favorable changes in gut microbiome composition
Meslier 2020 [47]	Eighty-two overweight and obese subjects with low fruit and vegetable intake	MD	Habitual diet	8 weeks	↑ *Faecalibacterium prausnitzii* ↓ *Ruminococcus gnavus* ↑ representation of genes involved in carbohydrate metabolism and SCFA synthesis	↓ plasma cholesterol ↑ Insulin sensitivity ↓ fecal bile acids	MD resulted into increased levels of urinary urolithins, proportionate to plant food intake
Pagliai 2020 [48]	Twenty-three overweight subjects with low/moderate cardiovascular risk	Low-calorie MD	Vegetarian diet	3 + 3 months (cross-over design)	↑ *Enterohabdus, Lachnoclostridium, Parabacteroides* ↑ synthesis of propionic acid	↓ inflammatory cytokines	No major changes in microbiome composition with either dietary pattern
Rinott 2022 [49]	Two hundred and forty-nine subjects with abdominal obesity/dyslipidemia	MD or Green MD plus walnuts	Standard guideline-based healthy diet	6 months	↑ *Prevotella* ↓ *Bifidobacterium* ↑ representation of genes involved in branched-chain amino acid metabolism	↓ Body weight, blood pressure, blood lipids ↑ Insulin sensitivity	The beneficial effects of MD are mediated by specific changes in gut microbiome, that are emphasized in green MD
Calabrese 2022 [50]	One hundred and nine patients with NAFLD	LGIMD with or without aerobic activity	Standard guideline-based healthy diet	3 months	Changes in overall composition ↑ *Ruminococcus, Enterohabdus, Coprobacter, Lachnospiraceae*	↓ elastosonographic index of liver fibrosis	The effects of MD on gut microbiome are emphasized when MD is associated with physical activity
Ben-Yacov 2023 [53]	Two hundred adults with pre-diabetes	MD	PPT diet based on machine-learning	6 months	↑ *Bifidobacterium adolescentis* and other taxa	↓ plasma cholesterol ↑ Insulin sensitivity	PPT diet was more effective in driving changes in gut microbiome composition
Haro 2016 [54]	Two hundred and thirty-nine patients with CHD (with or without metabolic syndrome)	MD	Low-fat diet	2 years	↑ *Parabacteroides distasonis, Bacteroides thetaiotamicron, Faecalibacterium prausnitzii, Bifidobacterium adolescentis* in patients with metabolic syndrome	↓ waist circumference ↓ glucose plasma levels ↓ triglycerides ↑ HDL	Mediterranean diet-induced marked changes in fecal microbiome composition only in subjects with metabolic syndrome
Galié 2021 [55, 56]	Thirty-eight overweight patients with metabolic syndrome	MD	Free diet with 50 g/day nut supplement	2 + 2 months (cross-over design)	↑ *Lachnospiraceae, Ruminococcaceae*	↓ glucose, insulin, HOMA-IR	MD-induced changes in microbiome were associated with positive changes in fecal acetate and with changes in 65 serum metabolites
Lewis 2021 [57]	One hundred and ninety-one patients with Crohn’s disease	MD	Carbohydrate diet tailored to Chron’s disease	6 weeks	No major differences in microbiome composition	Improvement of parameters of inflammation in both diets	Two distinct clusters of microbiome response to diet were identified, one with increasing abundance of *Bacteroides vulgatus* and the other with decreasing abundance of *Faecalibacterium prausnitzii*

*MD* Mediterranean Diet; *LFHCC* Low-Fat High-Complex Carbohydrate; *LGIMD* Low-Glycemic Index Mediterranean Diet; *PPT* Personalized Postprandial Targeting; *CHD* Coronary Heart Disease; *HDL* High-Density Lipoprotein; *HOMA-IR* Homeostatic Model Assessment of Insulin Resistance; *NAFLD* Non-Alcoholic Fatty Liver Disease

In a group of 20 healthy male volunteers, Barber et al. showed no major differences in gut-microbiome composition before and after a 2-week MD intervention, but they just demonstrated significant variations in a few bacterial taxa [41]. These findings were probably influenced by the high baseline inter-individual variability in the intestinal microbiome of participants, who responded to the dietary intervention in an individualized manner. However, metabolomic analyses revealed a different urinary metabolic profile after the intervention, with significant variations in the levels of several metabolites of bacterial origin [41]. In another study by Godny et al., a 4-week MD intervention associated with daily physical activity measurement resulted into increased representation of bacterial taxa involved in SCFA synthesis, including *F. prausnitzii, Lachnospiraceae* and *Bifidobacterium* spp., with reduced levels of inflammatory biomarkers such as fecal calprotectin [42]. Interestingly, similar results were obtained also by Rejeski et al. in a group of 10 healthy subjects, where a 2-week MD intervention also improved the overall microbiome species richness [43].

The modulatory effects of MD on gut microbiome are potentially useful for the prevention and treatment of several chronic conditions, including obesity, type 2 diabetes, and metabolic syndrome [44]. In 18 overweight subjects with body mass index (BMI) ≥ 25 kg/m^2^, a 3-month dietary intervention consisting of MD enriched with 40 g/day of high-quality extra-virgin olive oil was associated with improved fecal microbiome biodiversity, significant reduction of myeloperoxidase activity, oxidative stress markers and pro-inflammatory cytokines, and significant increase in adiponectin and plasma anti-inflammatory cytokines such as interleukin-10 (IL-10) [45].

Randomized controlled trials (RCTs) investigating the effects of MD and its variants on the gut microbiome of overweight and obese subjects have also shown beneficial effects, ranging from increased biodiversity to increased representation of SCFA-producing taxa or bacteria involved in branched-chain amino acid metabolism (Table 2) [46–49]. Interestingly, other healthy dietary patterns not falling within the MD definition, such as the vegetarian [48] or the low-fat high-complex carbohydrate (LFHCC) diet [46], also induced beneficial changes in gut-microbiome composition and function, but these changes were distinct from those induced by a traditional MD pattern. LHFCC diet, for example, determined a more pronounced increase in the representation of *F. prausnitzii* than MD [46], while vegetarian diet promoted growth of *Anaerostipes, Streptococcus, Clostridium,* and *Odoribacter* not observed in MD [48].

The effects of MD on the gut microbiome are emphasized by concomitant involvement in a structured physical exercise program. In a group of subjects with non-alcoholic fatty liver disease (NAFLD), a condition frequently overlapped with obesity, the abundance of *Ruminococcaceae, Oscillospiraceae* and *Lachnospiraceae* was much more influenced by the association between aerobic physical exercise and MD than MD alone [50].

The interactions between gut-microbiome modifications induced by MD and type 2 diabetes are less known. In subjects following a MD pattern, an intestinal microbiome signature consisting in high abundance of *Prevotella, Saccharibacteria*, and *Betaproteobacteria* was associated with increased risk of developing diabetes [51]. Conversely, a 4-week MD intervention in subjects with type 2 diabetes resulted into an improved bacterial richness of gut microbiota, which exhibited a negative correlation with insulin resistance [52]. However, in a recent RCT comparing the effects of MD with a personalized post-prandial targeting (PPT) diet in subjects with type 2 diabetes (Table 2), MD was associated with only minor modifications of fecal microbiome, such as increased abundance of *Bifidobacterium adolescentis*, and resulted less effective than PPT diet in inducing metabolically-favorable changes of intestinal microbial communities [53]. Nevertheless, it should be highlighted that here the authors compared two different dietary strategies (general vs. personalized dietary advice) and that further interventions with personalized MD intervention could be of interest.

In subjects with metabolic syndrome (Table 2), however, a long-lasting MD intervention was associated with increases in the fecal microbiome representation of *Parabacteroides distasonis, Bacteroides thetaiotaomicron, F. prausnitzii, B. adolescentis* and *Bifidobacterium longum* [54]. In two different analyses of the METADIET randomized, controlled, cross-over trial, Galié et al. showed that MD intervention was associated with increased levels of *Lachnospiraceae* and *Ruminococcaceae* and improved insulin sensitivity in patients with metabolic syndrome (Table 2) [55, 56].

The effects of MD interventions on the gut microbiome of adult patients with other chronic illnesses not included in the metabolic syndrome spectrum have been scarcely investigated (Table 2). In a RCT comparing MD with the specific carbohydrate diet in adults with Crohn’s disease, neither dietary approach was superior in reducing parameters of inflammation and disease activity after 6 weeks, and no major differences in post-intervention fecal microbiome composition were detected between study arms [57]. Therefore, the current knowledge on the physiologic effects of the interaction between MD and intestinal microbiome in subjects younger than 70 years old is basically limited to improvements in insulin resistance, glucose and lipid metabolism, and chronic subclinical inflammation related to obesity (Table 2).

## Mediterranean diet and gut microbiome in older subjects

### Observational studies

Despite the gut-microbiome composition of older individuals is less resilient to stressors and more influenced by a large number of (physio)pathologic conditions, diet remains a major driver of the inter-individual variability observed for this age group. In a cross-sectional analysis of data from the NU-AGE RCT, including 226 Dutch subjects aged between 65 and 79, BMI exhibited a much stronger statistically significant association with overall fecal microbiome composition than frailty phenotype [58]. The main dietary factors explaining microbiome inter-individual variability were, on the one side, consumption of fruits, nuts, grain products, and carbohydrates, which are fundamental parts of the MD pattern, and, on the other side, consumption of processed and red meats, which are consumed only occasionally in MD [58]. The main bacterial taxa showing positive associations with foods typically well represented in MD included *F. prausnitzii, E. rectale* and *Eubacterium biforme*, while animal protein-based diets were mainly associated with the abundance of taxa with purported pro-inflammatory action, such as *Ruminococcus gnavus* and *Collinsella* [58].

Adherence to MD was associated with increased representation of *F. prausnitzii* also in a group of 74 older Spanish volunteers, where *Clostridium* cluster XIVa was identified as another important marker of MD [59]. These features of gut microbiome were also associated with specific metabolic signatures, including increased fecal levels of SCFAs, benzoic and 3-hydroxyphenylacetic acids [59, 60]. These signatures are likely the result of the increased-intestinal microbial metabolism of fibers and (poly)phenols associated with MD, respectively [59, 60]. In a group of older Caribbean Latinos living in the United States, instead, adherence to MD was associated with a distinct cluster of fecal microbiome composition, characterized by different abundance of *P. copri* and co-occurring bacterial networks [61].

In a group of 17 centenarians and 29 nonagenarians from Sardinia (Italy), adherence to MD was associated with several bacterial taxa in fecal microbiome [62]. However, the taxa positively correlated with MD were different between centenarians (*Lactobacillus, Clostridium,* and *Dorea*) and nonagenarians (*Bacteroides, Parabacteroides,* and *Pedobacter*), with the only exception of *Peptoniphilus* [62]. Interestingly, MD was associated with depletion of bifidobacteria only in nonagenarians, but not in centenarians [62]. In fact, the microbial ecology of centenarians is characterized by the persistence of a subdominant fraction of bifidobacteria, which is usually depleted in older individuals who do not reach extreme ages [63, 64].

### Intervention studies

The existing intervention studies with MD in older individuals have adapted the traditional MD pattern to the specific needs of aging, targeting, in particular, modulation of inflammation, weight loss, and cognition. Berendsen et al. elaborated a modified MD pattern based on recommended daily allowances (RDAs) of macro- and micronutrients for older subjects, with the aim of modulating inflammaging (i.e., the chronic subclinical activation of inflammatory pathways responsible for the pathogenesis of several chronic illnesses and geriatric syndromes typical of the older age) and maintaining a good balance in protein-energy metabolism [65, 66]. This dietary approach —the so-called NU-AGE diet— was the main intervention studied in the NU-AGE RCT, where 1141 pre-frail and fit subjects aged 65 to 79 years old from five European countries were randomized to receive MD tailored to older people (NU-AGE diet) or a control free diet [65]. It was demonstrated as feasible in pre-frail and non-frail older subjects in the long term, with good adherence scores after 1 year from the initiation of the intervention [67]. The gut-microbiota composition and function was among the endpoints of the NU-AGE study and fecal microbiome profiling of 612 participants showed significant changes in the abundance of several taxa [68]. *F. prausnitzii, E. rectale, Roseburia, Blautia, Anaerostipes* and *Prevotella* were the main taxa showing positive association with the NU-AGE dietary intervention, while the abundance of *Collinsella, Ruminococcus, Dorea, Blautia* and *Coprococcus* exhibited a negative association [68]. Interestingly, after 1-year intervention, those subjects with deeper degrees of gut-microbiome change, reflected in higher levels of the MD microbiome index, also exhibited significant improvement in measures of inflammation (CRP levels), frailty (Fried score and gait speed) and cognition (verbal fluency, Babcok memory score, and constructional praxis score). Conversely, the subjects in the intervention arm who exhibited reduced variations of fecal microbiome composition experienced less pronounced variations in clinical endpoints [68]. Therefore, the NU-AGE diet intervention modulated gut microbiota in a way that reflected negative associations with inflammation and frailty.

A MD-based intervention with energy restriction resulted into significant modulation of fecal microbiome even after only 15 days in a smaller study conducted in 20 obese older women [69]. However, the observed changes were partly different than those of the NU-AGE RCT, with increased representation of *Coprococcus, Oscillospira, Bacteroides*, and *Akkermansia*, while *Collinsella* was confirmed as negatively associated with the dietary intervention [69]. In the PREDIMED-Plus Study, traditional MD intervention was compared with an energy-restricted MD associated with promotion of physical activity in a group of 343 overweight and obese Spanish subjects aged between 55 and 75 years old [70]. Interestingly, the changes observed in gut microbiota after 1-year follow-up were similar between the two groups, but more pronounced in the energy-restricted MD arm, and included increased representation of SCFA producers from the *Lachnospiraceae* family [70].

The effects of MD intervention approaches on intestinal microbiome have been recently studied also in age-related neurological conditions. Rusch et al. showed significant changes in fecal microbiome composition, including in particular an increase in the SCFA producer *Roseburia*, after a 5-week MD intervention in a small group of patients with Parkinson’s disease [71]. These changes were associated with improvements in gastrointestinal symptoms (constipation scores), but no neurologic endpoints were measured in the study.

In the field of dementia research, ketogenic diets have shown a potential of slowing down cognitive impairment by improving cerebral metabolism [72]. Recently, some authors have proposed to combine the Mediterranean and ketogenic diet approaches into a modified Mediterranean-ketogenic diet (MMKD) [72], which has shown the capacity of improving body composition and reducing levels of cerebrospinal fluid biomarkers of amyloid deposition (Aβ42) and tau protein [73, 74]. In a randomized cross-over trial conducted on 17 subjects with and without mild cognitive impairment, 6-week MMKD intervention induced increased representation of *Akkermansia* and *Slackia* and reduced *Bifidobacterium* and *Lachnobacterium*, with the family *Lachnospiraceae* showing significant correlation with cerebrospinal fluid levels of Aβ42 [75]. Interestingly, changes in symbiont intestinal fungal species, the so-called mycobiome, were also observed [76]. At the functional level, MMKD induced changes in bacterial SCFA synthesis, with significant increases in fecal butyrate and propionate levels [75]. In a recent study conducted in 20 subjects with mild cognitive impairment, MMKD induced lower representation of *Alistipes*, a bacterial species known for its capacity of producing gamma-amino-butyric acid (GABA), and higher representation of *Akkermansia*, a taxon with GABA-regulating functionality [77]. GABA imbalance is involved in the gut-brain axis and in the pathogenesis of dementia. Therefore, MD diet could influence important aspects of cognitive function by inducing subtle modifications in gut microbiome.

## Anti-Aging effects of mediterranean diet mediated by intestinal microbiome: main underlying mechanisms

### Microbial synthesis of short-chain fatty acids (SCFAs)

The depletion of bacterial taxa with capacity to synthetize SCFAs, and particularly *F. prausnitzii, Roseburia, Butyrivibrio*, and *Succinivibrio*, is a keynote characteristic of the gut microbiome of older individuals with frailty [78–80], sarcopenia [81, 82] and cognitive decline [83]. Frailty is associated with reduced fecal levels of butyrate [80], and an inverse correlation has been demonstrated between gut microbial synthesis of butyrate and appendicular lean mass in subjects at risk for sarcopenia [82]. Conversely, fit centenarians generally exhibit higher fecal levels of SCFAs than subjects between 60 and 70 years old [84].

In this scenario, the capacity of MD to stimulate the growth of SCFA-synthetizing bacteria and improve SCFA levels can be extremely important in an anti-aging perspective. However, the functional capacity of gut bacteria to effectively release SCFAs does not depend only on the fiber content of diet, but it also relies on complex cross-feeding interactions among bacteria and on the interaction between bacteria and host [85, 86]. For example, an adequate butyrate production by *F. prausnitzii* is possible only in presence of a good representation of bifidobacteria in the gut environment [87, 88].

SCFAs, particularly butyrate, exhibit pleiotropic functions for the host [89]. First, they promote gut mucosal integrity and tropism, representing one of the main sources of energy for colonocytes [89]. Second, they exhibit marked capacity to modulate the inflammatory response, which is pivotal for controlling inflammaging and the pathogenesis of several age-related diseases and conditions, including frailty [90, 91]. In a group of Chinese older patients with cognitive impairment related to diabetes, reduced fecal butyrate levels were associated with higher circulating levels of pro-inflammatory cytokines and worse cognitive performance [92]. In animal models of dementia, butyrate administration is in fact able to improve indices of neuroinflammation and cognitive performance via the gut-brain axis [93, 94].

In systemic circulation, butyrate also improves insulin resistance and has an overall pro-anabolic function, which is pivotal in counteracting type 2 diabetes and obesity [95]. At the skeletal muscle level, these actions result into increased protein synthesis and reduced muscle wasting [96], and this is the main reason why butyrate is considered a powerful anti-sarcopenic mediator in the context of the so-called gut-muscle axis [82, 97]. In fact, butyrate also exhibits an inhibitory capacity at the histone deacetylase level, resulting into increased muscle mass in mouse models of cachexia [98].

### Reduction of intestinal mucosa permeability

Aging, even with a healthy active pattern, is associated with increased-intestinal permeability, witnessed by elevated levels of the serum biomarker zonulin [99]. According to a recent meta-analysis of case–control studies investigating biomarkers of frailty, serum zonulin levels are in average higher in frail than in healthy older subjects, reflecting a progressive loss of the barrier function of the intestinal mucosa [100]. This condition is associated with loss of skeletal muscle strength, sarcopenia, and functional autonomy in older subjects, either healthy or with chronic conditions such as chronic obstructive pulmonary disease (COPD) and dementia [101–103].

Increased intestinal mucosa permeability is associated with increased serum levels of bacterial toxins, including lipopolysaccharide (LPS), and increased presence of bacterial components into systemic circulation [104, 105]. These compounds provide activation of the innate immune response and antigenic stimulation of adaptive immunity, that ultimately result into persistent subclinical inflammation typical of aging with frailty, the so-called inflammaging [106]. Increased LPS toxinemia plays a pivotal role in the pathophysiology of age-related cognitive decline and Alzheimer’s disease, and is believed to represent one of the mainstays of the gut-brain axis dysregulation [107–109]. Age-related gut-microbiome alterations are deeply involved in this pathophysiological cascade, since germ-free mice show no development of chronic inflammation during aging and no increased LPS levels, while old mice with dysbiosis exhibit increased circulating levels of pro-inflammatory cytokines and macrophage dysfunction [110].

Higher adherence to MD is inversely associated with biomarkers of gastrointestinal mucosa permeability and with circulating LPS levels, in both adult subjects with chronic illnesses and older individuals [105, 111, 112]. These effects are emphasized with dietary interventions consisting in increased intake of (poly)phenol-rich foods, which are important components of MD [104, 113, 114]. In particular, the MaPLE randomized controlled trial showed that a (poly)phenol-rich food intervention in older subjects caused reduction in the serum levels of zonulin, associated with favorable changes in the gut microbiome including the increase of the relative abundance of *F. prausnitzii* [113]. Interestingly, these effects were less pronounced in those subjects with higher disruption of gut microbial community structure and with increased-intestinal permeability at baseline [104, 114]. Dysbiosis, in fact, does not only impair the integrity of the intestinal mucosa, but it can also limit the bioavailability of food bioactives contained in MD, especially (poly)phenolic compounds [115], making dietary interventions probably less effective in subjects with higher burden of frailty and associated gut-microbiome alterations.

The precise interactions among components of MD, gut microbiome, and host cells regulating intestinal mucosa permeability have not been elucidated yet. However, recent studies suggest that SCFAs and the bacterial taxa able to synthetize them from dietary fibers may play a central role [116, 117]. In the LIBRE RCT, for example, the baseline levels of SCFAs were independent predictors of the response to MD intervention in terms of intestinal permeability [117]. Therefore, the individual responses to MD diet may be consistently mediated by the preexisting composition and functionality of gut microbiome.

### Biotransformation of food bioactives

(Poly)phenols or phenolic compounds are generally abundant in MD, where the intake of fruit and vegetables, wholegrain cereals, nuts, legumes, and extra virgin olive oil is recommended in large amounts [118]. The interaction between dietary (poly)phenols and the gut microbiome is able to generate several bioactive metabolites exhibiting anti-aging effects, especially at the skeletal muscle and central nervous system level [86, 119, 120]. However, the microbiome response to dietary interventions shows a consistent inter-individual variability in terms of production of bioactive compounds [121, 122]. In the case of some polyphenolic subclasses, including ellagitannins, flavanones, isoflavones, flavan-3-ols, prenylflavonoids, avenanthramides, resveratrol, and lignans, some microbiota-related metabotypes have been identified, so that the beneficial effects of these dietary components might be observed only in presence of a particular microbiome composition and functionality [121, 122].

For example, urolithin A, isourolithin A, and urolithin B are metabolites released by the gut microbiome after ingestion of ellagic acid and ellagitannins, polyphenols frequently found in walnuts, pomegranate, and strawberries. Not all the individuals are able to produce these metabolites, and subjects can be classified into the Uro-A or Uro-B metabotype according to their capability to not produce or produce, respectively, urolithin B or isourolithin A, in addition to urolithin A [123]. Conversely, subjects with the Uro-0 metabotype do not show production of (iso)urolithins A or B by the gut microbiome, even after a dietary challenge with foods with high ellagitannin content, and cannot benefit from the biologic functions of urolithins [123]. While isourolithin A and urolithin B have been associated to gut dysbiosis and the prevalence of the Uro-B metabotype increases with aging, urolithin A production and the Uro-A metabotype has been related to a healthier and younger profile [122]. The potential anti-aging effects of urolithin A include improvement of muscle strength and exercise endurance [124, 125], modulation of neuroinflammation and cell apoptosis with improvement in cognitive function [126–128], promotion of insulin sensitivity, modulation of lipid metabolism and inflammatory response [129]. Interestingly, in a recent RCT testing the effects of MD in obese subjects, MD was associated with an average increase in the urinary excretion of urolithins, even if the analyses did not consider metabotypes [47]. Similarly, urolithin urinary excretion was significantly associated with visceral adiposity reduction [130] and magnetic resonance-measured hippocampal occupancy score [131] in two distinct RCTs testing the effects of a long MD intervention.

Gut microbiota-derived metabotypes are less known for polyphenol subclasses other than ellagitannins. Hesperidin high-excretors and low-excretors have been identified after dietary intake of flavanones, a polyphenol subclass particularly represented in citrus [121, 122]. Hesperidin exhibits antioxidant, anti-inflammatory and pro-anabolic actions, promoting muscle protein synthesis [132, 133] and reducing amyloid deposition and neuroinflammation in animal models of Alzheimer’s disease [134, 135]. In an intervention study testing the effects of MD in subjects with type 2 diabetes, increased plasma levels of hesperidin and other flavanone derivatives were detected after 12 weeks, with significant reductions in inflammatory biomarkers [136]. Similarly, equol is a bioactive compound released after intestinal biotransformation of soy isoflavone daidzein, but it is produced only by a part of the population harboring a specific microbial profile. Equol exhibited neuroprotective actions against the onset of dementia in vitro [137–139], but it was associated in vivo with better cognitive performance only in presence of an equol-producer microbiome metabotype [140].

## The role of dietary proteins and exercise in older age: a gut microbiome perspective

Nutritional guidelines and clinical recommendations against age-related physical frailty and sarcopenia generally emphasize the importance of increasing protein intake to overcome anabolic resistance [141, 142]. However, intervention studies have shown only mild improvements in muscle mass and strength after increases of daily protein intake up to 1.6 g/kg/day [143]. High-protein diets, especially rich of processed foods of animal origin, are also associated with deleterious consequences for the gut-microbiome composition and functionality in both human and animal experiments [144]. A recent systematic review of RCTs conducted in human beings has shown that higher meat intake is associated with marked reduction of *Anaerostipes* and *Faecalibacterium*, two of the main taxa producing SCFAs [145]. In this perspective, ad libitum consumption of animal proteins in the older age may produce conflicting effects on the pathophysiological mechanisms of physical frailty and sarcopenia, resulting in modest clinical benefits [146, 147].

The benefits of high-protein diets on frailty and inflammation, instead, may be more pronounced when the dietary intervention consists in consumption of proteins with high biologic value, such as whey protein [148, 149], or when dietary intervention is associated with regular exercise [150]. In a double-blind placebo-controlled cross-over study, Ford and colleagues demonstrated that a balanced high-protein diet, either in combination with probiotics/synbiotics or alone, was associated with generally favorable changes in the gut-microbiota composition of a group of healthy older women, although the representation of the SCFA producers *Roseburia* and *Anaerostipes* was reduced [151]. In healthy young subjects, the increase of lean red meat consumption under controlled conditions was not associated with detrimental consequences for the microbiome structure as well [152].

Protein consumption in MD is balanced and mainly includes vegetal proteins of high biologic value, such as those from legumes and lean meat, rather than proteins from processed red meats. Thus, increasing protein intake maintaining a Mediterranean dietary style may represent an optimal compromise from a gut-microbiota perspective [146, 147].

Furthermore, regular exercise seems to act as an enhancer of the beneficial effects of MD-style diets, both on the gut-microbiome composition and on clinical markers of aging. Multicomponent interventions consisting in regular exercise and tailored nutritional counseling have proven effective in reducing the burden of disability in physically frail older individuals [153, 154]. Exercise represents a powerful beneficial modulator of gut microbiome and can prevent dysbiosis in both adult and older age [155, 156]. However, no comprehensive investigation of the effects of exercise and its interaction with dietary patterns has been conducted in older individuals to date. Interestingly, in the PREDIMED-Plus Study the effects of energy-restricted MD dietary interventions on the gut microbiome of subjects aged 55–75 years old were more pronounced when diet was associated with regular exercise programs [70]. Therefore, multicomponent interventions combining MD or its variants with exercise programs should represent the best alternatives to counteract age-related frailty also from a gut microbiome perspective. Future studies should include also gut-microbiome parameters as endpoints of clinical interventions against frailty.

## Conclusion and perspectives

**Fig. 1:**
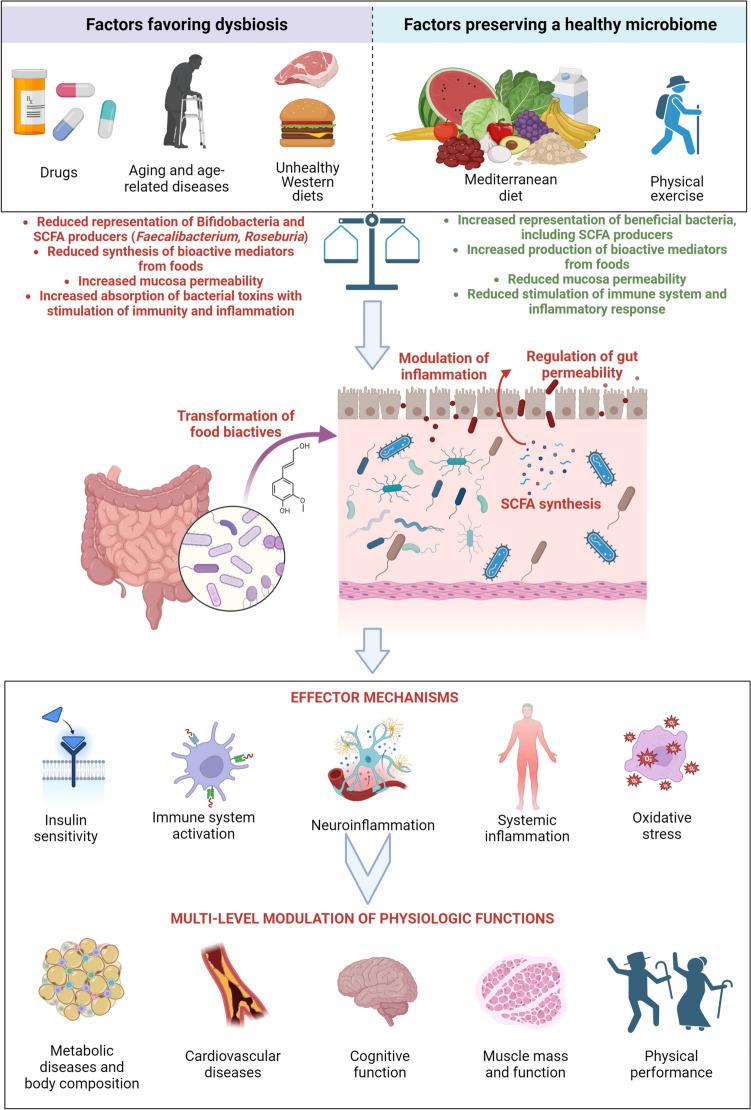
Overview of the mechanisms linking dietary habits, and particularly Mediterranean diet, to gut-microbiome and pathophysiological aspects of aging (created with Biorender.com)

A healthy diet can influence several pathophysiological aspects of aging through mediation of the intestinal microbiome, while Western-style diets may be associated with a tendency toward dysbiosis favoring the pathophysiological processes leading to frailty (Fig. 1). In this context, promoting MD in older individuals can represent an effective strategy to counteract the age-related drift of gut-microbiome composition and function toward dysbiosis and its detrimental consequences. In both adult subjects and older individuals, adherence to the MD pattern is associated with maintenance of a healthy microbiome able to modulate inflammation, anabolic resistance, oxidative stress, and neurodegeneration in a favorable way. Intervention studies have confirmed this interaction among diet, gut microbiome, and host (patho)physiology, but few studies have specifically targeted older individuals and outcomes of geriatric interest. In this regard, the results from the NU-AGE study, suggesting that older individuals respond to MD intervention in an individualized manner by mediation of the intestinal microbiome, are of paramount importance to understand the complex underlying mechanisms linking diet, aging, and its phenotype. Future studies should further investigate the role of MD and its variants in counteracting physical and cognitive decline in the older age, accounting also for the role of the microbiome from a multi-omics perspective.

## Data Availability

Data sharing not applicable to this article as no datasets were generated or analyzed in the present work.
